# Left ventricular cavity obliteration: Mechanism of the intracavitary gradient and differentiation from hypertrophic obstructive cardiomyopathy

**DOI:** 10.1111/echo.14710

**Published:** 2020-05-22

**Authors:** Charles Pollick, Hezzy Shmueli, Nicolas Maalouf, Ronit H. Zadikany

**Affiliations:** ^1^ Smidt Heart Institute Cedars Sinai Medical Center Los Angeles California USA

**Keywords:** aortic stenosis, hypertrophic cardiomyopathy, left ventricular function

## Abstract

**Background:**

Controversy surrounds the cause of the pressure gradient in patients with hypertrophic obstructive cardiomyopathy (HOCM). Left ventricular cavity obliteration (LVCO) was first described as the cause of the gradient but subsequently systolic anterior motion (SAM) of the mitral valve has been established as the cause. Nevertheless, the two gradients, though different in origin and significance, share similar characteristics. They both have a similar “dagger” profile, are obtained from the cardiac apex, are associated with a hyperdynamic left ventricle, and the gradients are worsened by Valsalva. The distinction has clinical relevance, because treating the intracavitary gradient (ICG) of LVCO as if it were a SAM‐associated gradient associated with HOCM would be inappropriate and possibly harmful.

**Materials and Methods:**

To clarify the cause and characteristics of the ICG in patients with LVCO in patients without HOCM, we assessed the extent and duration of cavity obliteration, and for differentiation, we compared the spectral profiles with patients with HOCM and severe aortic stenosis (AS).

**Results:**

Higher ICG is associated with a greater extent and more prolonged apposition of LV walls, and smaller left ventricular cavity size.

The spectral profile of patients with AS, HOCM, and LVCO is differentiated by the peak/mean gradient ratios of 2 or less, 2–3, and 3 or greater, respectively, in >90% of patients. Most patients with LVCO without HOCM or severe LVH have an ICG < 36 mm Hg.

**Conclusion:**

The magnitude of ICG is quantitatively associated with the extent and duration of LVCO. Spectral profiles of severe AS, HOCM, and LVCO can be differentiated by the peak/mean gradient ratio.

## 1 INTRODUCTION

Left ventricular cavity obliteration (LVCO), defined as obliteration of the apex in systole on angiography, was first described^1^ in 1965 and proposed as the cause of the intraventricular pressure gradient accompanying hypertrophic cardiomyopathy. It was subsequently documented^2^ that cavity obliteration can be seen in states other than hypertrophic cardiomyopathy. Another school of thought^3^ opined that the pressure gradient in hypertrophic cardiomyopathy was due to left ventricular outflow tract (LVOT) obstruction. Following decades of study, it is now generally agreed that the characteristic gradient in hypertrophic obstructive cardiomyopathy (HOCM) is a dynamic subaortic pressure gradient due to LVOT obstruction from systolic anterior motion of the mitral valve (SAM).^4^, ^5^ These LVOT gradients, when high, are accompanied by exercise intolerance, which can be mitigated by pharmacologic or interventional methods to ameliorate the gradient.^6^ At first, the intracavitary gradients (ICG) accompanying LVCO were dismissed as either not obstructive^1^ and therefore not important, or possibly an artifact of “catheter entrapment”,^3^ and therefore also not clinically relevant. Intracavitary gradients with cavity obliteration have been demonstrated during dobutamine stress echocardiography and have, paradoxically, been associated with favorable, rather than adverse, outcomes.^7^, ^8^ More recently, however, apical cavity obliteration has been associated with adverse outcomes and has been implicated in the pathogenetic mechanisms of apical aneurysm in hypertrophic cardiomyopathy_._ ^9^ Despite such studies focusing on cavity obliteration, there is a lack of data studying the temporal and quantitative relationship between the 2D echocardiographic occurrence of obliteration and the magnitude of the ICG.

In addition to the controversy between the mechanism and significance of the gradient associated with LVCO and that of HOCM, the two gradients may be confused for a variety of reasons. They share a similar “dagger” profile, the gradients are both obtained from the cardiac apex, both are associated with a hyperdynamic left ventricle, and the gradients are both worsened by Valsalva. In patients with challenging echocardiographic windows, it may not always be possible to distinguish the origin of the gradient. Furthermore, they can coexist in patients with HOCM. The distinction has clinical relevance, because treating the ICG gradient as if it were an LVOT gradient associated with HOCM would be inappropriate and possibly harmful.

## 2 METHODS

### 2.1 Patients

We studied the most recent 100 patients in our echocardiography laboratory database search with the phrase “cavity obliteration” (LVCO) entered on a transthoracic echocardiogram report. Out of those, there were 87 patients *without* severe valve disease, severe pulmonary hypertension (PA systolic pressure >65 mm Hg), hypertrophic cardiomyopathy (HCM) (nonobstructive or obstructive), significant LV hypertrophy (defined as 15 mm or greater wall thickness), or SAM (moderate or greater) with clearly defined spectral profiles of intracavitary gradients. Of these 87 patients, there were 65 patients (female 48; mean age 74: range 40–101) who also had a well‐defined, nonforeshortened, and quantifiable LV cavity demonstrated on apical 4‐chamber views. In all patients, cavity obliteration was defined as obliteration of the LV apical cap with variable extension into the mid‐LV cavity. Of these 65 patients, 49 were inpatients, 17 were on intravenous inotropes, and 5 patients had sepsis; there were a variety of other diagnoses including chest pain, pneumonia, sclerosing cholangitis, and GI bleed.

For comparison, the spectral profiles of 25 patients with HOCM without LVCO (peak gradient range 32‐105 mmHg, average peak gradient 66 mmHg, average mean gradient 29 mmHg) and severe systolic anterior motion of the mitral valve (SAM), and 25 patients with severe AS without LVCO (peak gradient range 38‐89 mmHg, average peak gradient 63 mmHg, average mean gradient 37 mmHg) were assessed and compared with the spectral profile associated with the ICG seen with LVCO in a subset of 25 of the 65 patients with intracavitary gradients of 36 mm Hg or greater.

### 2.2 Transthoracic echocardiography

Standard transthoracic echocardiographic (TTE) studies were performed, using standard American Society of Echocardiography guidelines,^10^ in all patients using a commercially available ultrasound system with phased array transducers (Philips Medical Systems).

### 2.3 Echocardiographic measurements

**FIGURE 1:**
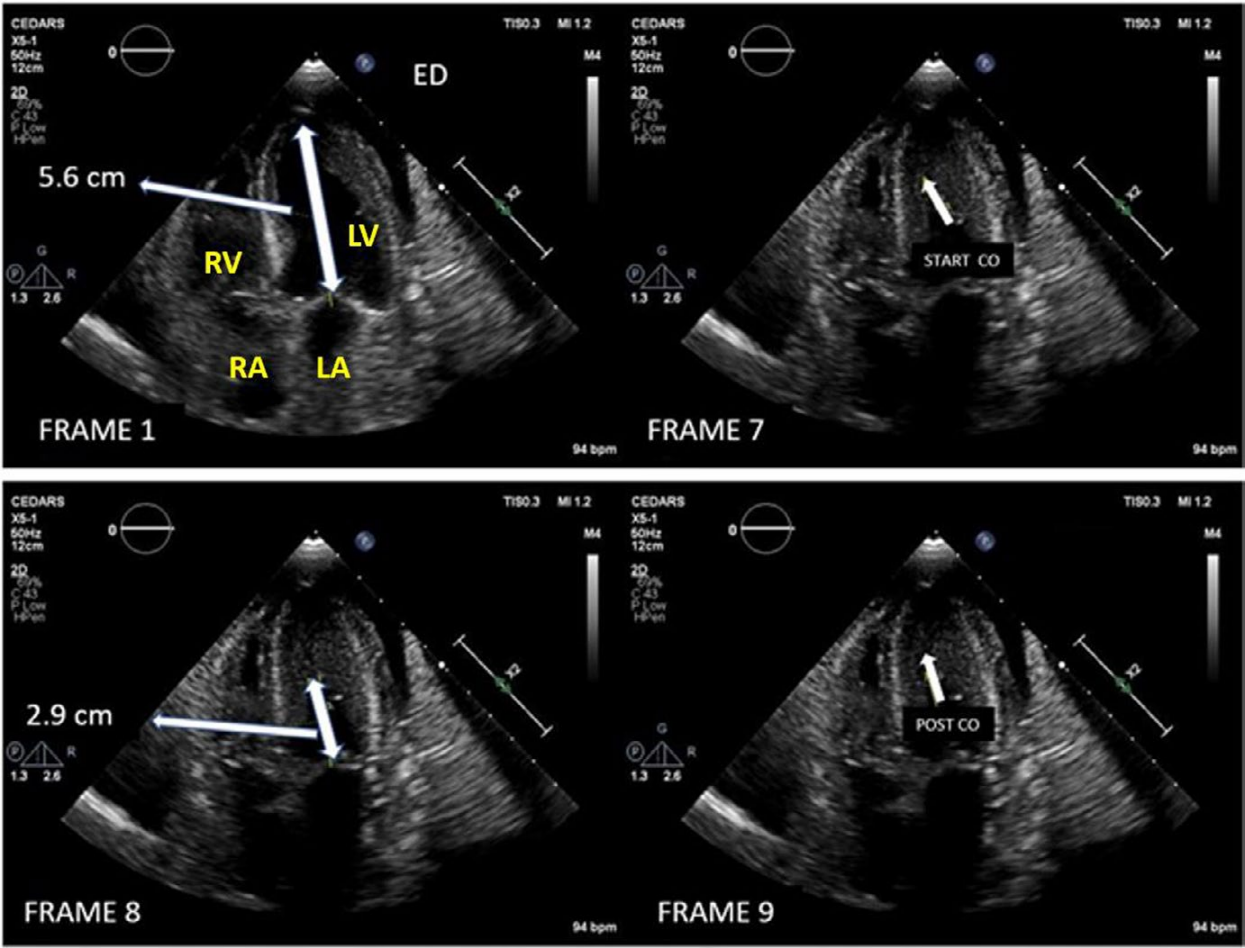
2D echo apical 4‐chamber frames in a LVCO patient with peak ICG of 2.1 mm Hg. End‐diastolic (ED) frame at top left. Apical length from apex to mitral annulus is 5.6 cm. Cavity obliteration first occurs in frame 7 and has already ended by frame 9 (post‐CO), which shows a tiny gap between the LV walls; therefore, only 2 frames; 7 and 8: are obliterated. At a frame rate of 50 Hz, this equals 20 ms per frame, and therefore, obliteration lasts 2 x 20 ms, or 40 ms. End obliteration apex to annulus length in frame 8 is 2.9 cm. Percent obliteration = 5.6‐2.9/2.9 = 48%

In the standard apical 4 (Ap4)‐chamber view (Figure 1), the following measurements were made from one clear representative cardiac cycle: (a) The end‐diastolic length (Ap4d) of the left ventricle from apex endocardium to the mitral annulus (mm); (b) the length of the obliterated cavity (Ap4s) from the most basal point of obliteration to the annulus (mm); and (c) the number of frames during which the LV cavity was obliterated was converted into msec. Frame duration was calculated from the frame rate in Hz. For example, if the frame rate was 50 Hz, this means that each frame is 20 ms. If the LV cavity was obliterated for 4 frames at 50 Hz, then that translates to 80 ms of obliteration. Measurements were made by 2 observers blinded to the ICG mm Hg measurements.

### 2.4 Doppler measurements

For the LVCO patients, the intracavitary spectral continuous‐wave Doppler profiles were identified. Peak and mean gradients were measured from one clear representative cycle. For the HOCM and AS patients, one clear representative spectral aortic and LVOT spectral profile was identified, and the mean and peak gradients were measured. Measurements were made by 4 observers blinded to the 2D measurements.

### 2.5 Statistical methods

Standard *t* tests for unpaired variables were performed. Standard Pearson's correlation coefficients were determined.

Permission to access the echocardiographic images and patient data was approved by our institutional review board.

## 3 RESULTS

### 3.1 Intracavitary gradients—range, shape, and correlation with apical 4‐chamber echocardiographic measurements

**FIGURE 2:**
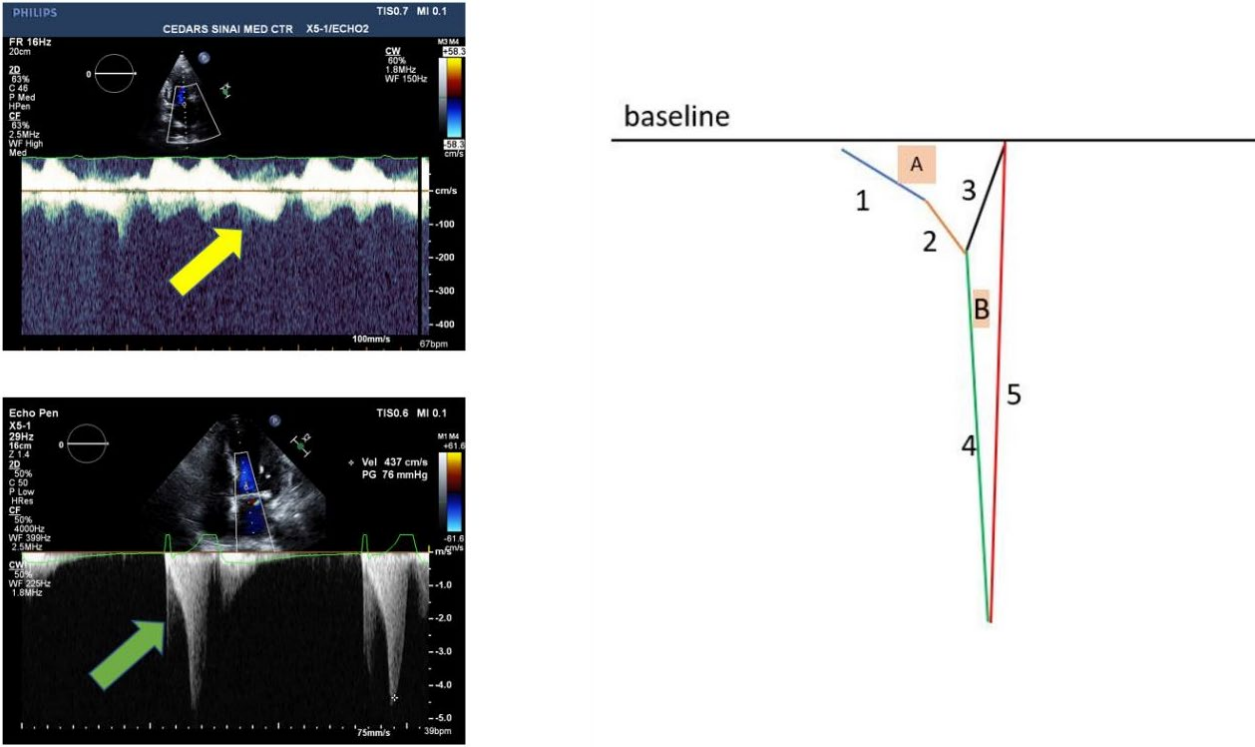
Left panel: upper—typical spectral profile in a patient with a small peak ICG (yellow arrow); lower—typical spectral profile in a patient with a higher peak ICG (green arrow). Right panel: schematic of the spectral profile in a patient with a small ICG gradient (A) with an initial slow acceleration (1) that speeds up (2) and then decelerates (3). For the higher gradients (B), the acceleration continues (4) to a peak and then rapidly decelerates (5)

**FIGURE 3:**
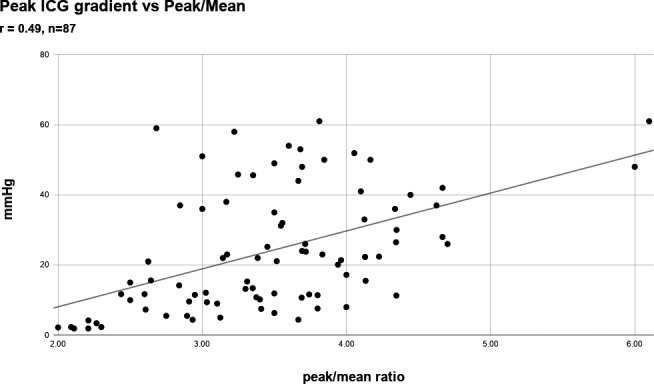
Peak ICG gradient vs peak/mean ratio for all 87 patients

In 62 of 87 patients with clearly definable intracavitary gradients, the gradient was 35 mm Hg or less (14 ± 10 mm Hg: range 2–31 mm Hg) vs 36 mm Hg or more (44.3 ± 12 mm Hg: range 36–61 mm Hg) in the remaining 25 patients. The shape of the spectral profile in patients with smaller gradients looked different from those with higher gradients (Figure 2). As the gradient increased, the ratio of peak/mean gradient concomitantly rose (*r* = 0.49; *P* < .0001) (Figure 3). Of the 62 patients with a gradient of 35 mm Hg or less, the peak/mean gradient ratio was 3.3 (SD 0.68) vs 3.85 (SD 0.85) for the patients with gradients of 36 mm Hg or higher (*P* = .007).

**FIGURE 4:**
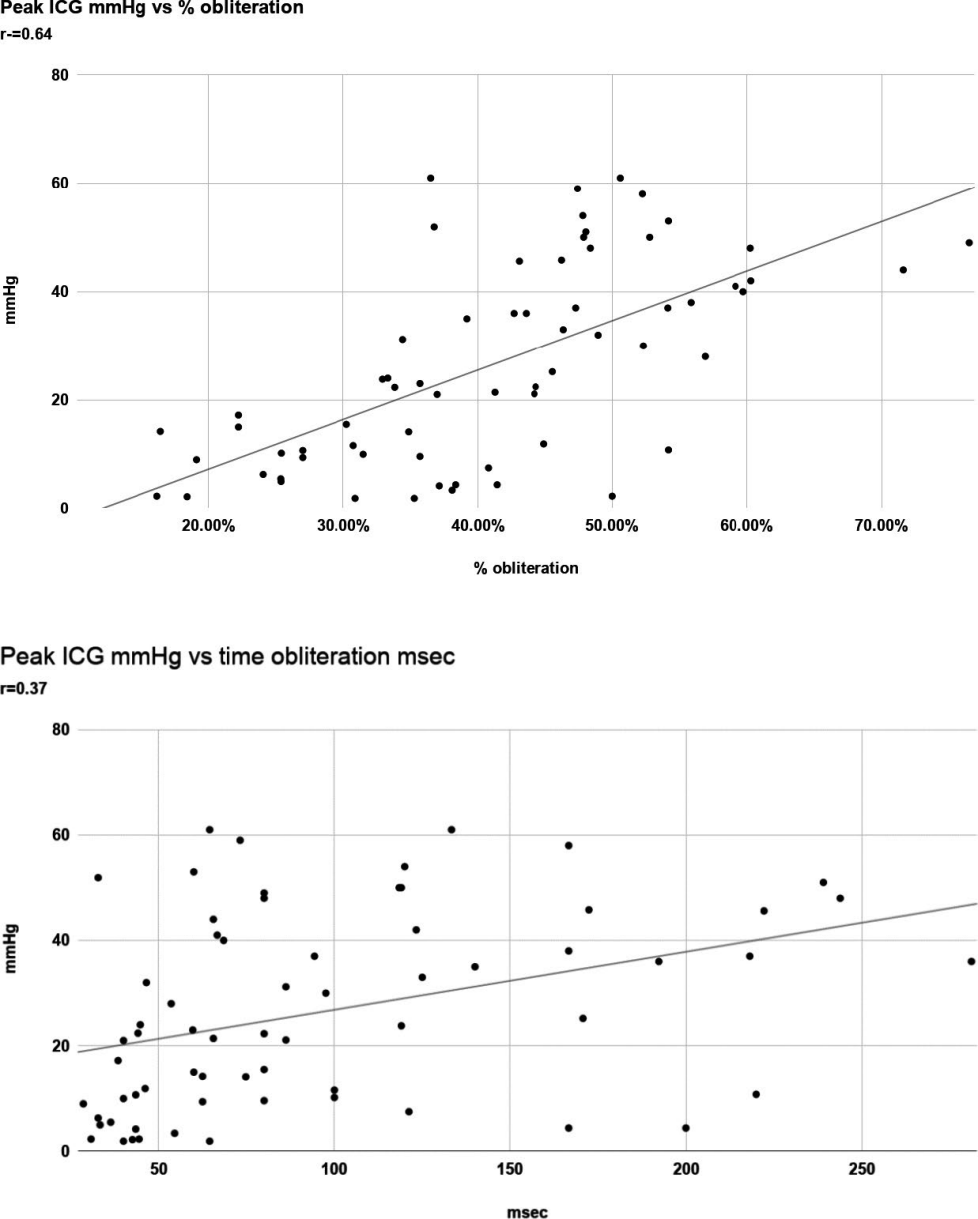
Upper plot: peak ICG vs percentage obliteration in 65 patients; lower plot: peak ICG gradient vs time in obliteration in 65 patients

**FIGURE 5:**
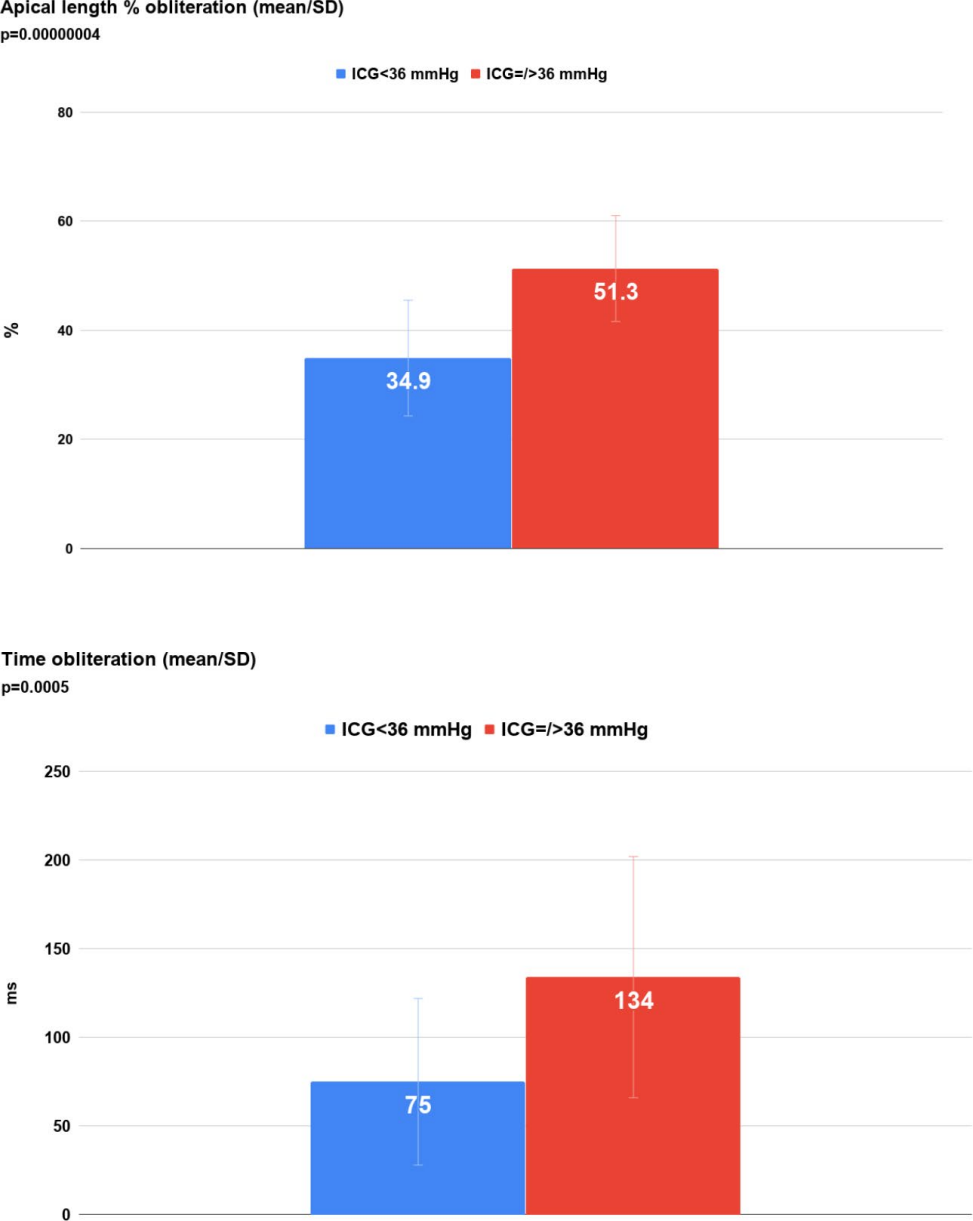
Upper panel: apical length % obliteration in patients subgrouped into peak gradients <36 mm Hg and ≥36 mm Hg; lower panel: time in obliteration in patients subgrouped into peak gradients <36 mm Hg and ≥36 mm Hg

In the 65/87 patients with clearly quantifiable LV cavity on apical 4‐chamber views, there was a positive correlation between the magnitude of the peak ICG (mm Hg) and the extent of cavity obliteration expressed as a percentage of the end‐diastolic length minus the end‐systolic length divided by the end‐diastolic length: *r* = 0.64 (*P* < .0001) (Figure 4). The difference was more obvious when comparing the 40 patients with 35 mm Hg or less gradient vs the 25 patients with 36 mm Hg or more (34.9% vs 51.3%, respectively) (*P* < .0001; Figure 5). The magnitude of the ICG also correlated, as a group, although weakly, with the duration of obliteration: *r* = 0.37 (*P* < .001; Figure 4). The difference in time of apposition was highlighted, however, by comparing the 40 patients with 35 mm Hg or less gradient vs the 25 patients with 36 mm Hg or more (mean 75 ms vs mean 134 ms, respectively (*P* = .0005)), indicating that the group of patients with higher gradients have more prolonged apposition than the group with smaller gradients (Figure 5). Comparing the baseline and echocardiographic data between the 40 patients with ICG of 35 mm Hg or less vs the 25 patients with ICG gradient of 36 mm Hg or more, there was no significant difference in age (74 ± 14 vs 76 ± 14), gender (F 26/40 vs F 21/25), or the echocardiographic parameters of interventricular septal thickness (12 vs 11 mm), posterior wall thickness (11 vs 11 mm), EF (76% vs 77%), left atrial area (18.5 vs 17.0 cm2), left ventricular outflow tract velocity time integral (30.2 cm vs 29 cm), or PA systolic pressure (33 vs 39 mm Hg). There was a significant difference in 2D derived left ventricular end‐diastolic cavity M‐mode dimension (37 vs 33 mm [*P* = .02]) and left ventricular end‐systolic cavity M‐mode dimension (23 vs 21 mm [*P* = .03]), indicating the left ventricular cavity size was smaller in the patients with ICG of 36 mm Hg or higher.

### 3.2 Qualitative and quantitative comparison of systolic velocity profiles between LVCO, HOCM, and aortic stenosis

**FIGURE 6:**
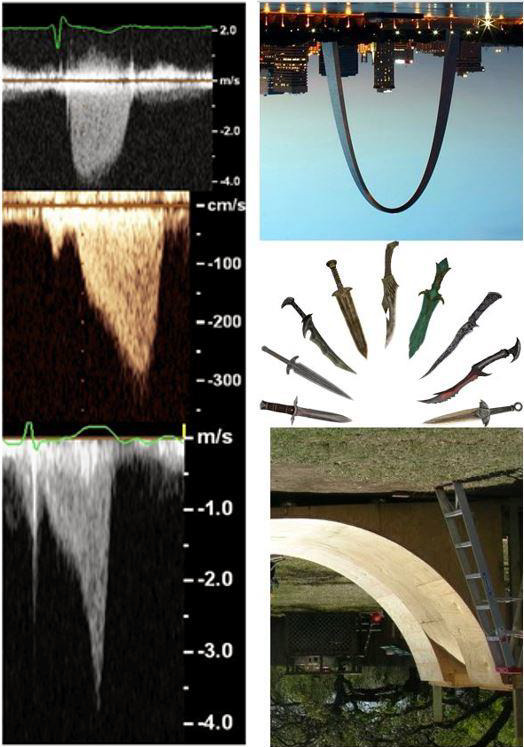
Left panel: AS (upper), HOCM (middle), and LVCO (lower) profiles. Right panel: real‐life images of a parabola (inverted city arch), daggers, and inverted half‐pipe skateboard ramp

*Qualitatively*, the LVCO ICG spectral Doppler profile has a similar profile to the HOCM Doppler profile (Figure 6) and hence may be confused. The HOCM LVOT initial acceleration is slow followed by a second phase of acceleration, which is faster as also seen with the spectral profile of the ICG associated with LVCO. In the LVCO patients with higher gradients, however (Figure 2), the second phase of acceleration appears steeper and faster than seen with the profile of the HOCM‐associated LVOT gradient. Indeed, the second phase of acceleration of the ICG spectral profile appears almost exponential and can be compared to one side of an inverted half‐pipe skateboard ramp (Figure 6).

To *quantify* the difference between the profiles, we assessed the ratio between peak and mean gradients. Consistent with the exponential, scooped‐out appearance of the second acceleration portion of the profile, the ratio of peak to mean gradient was significantly higher in the LVCO than HOCM patients: 3.5 (range 2.0–6.1) vs 2.4 (range 1.8–3.25), respectively (*P* < .0001). The difference between LVCO and HOCM peak/mean gradient ratios was even more marked when comparing the 25 patients with LVCO ICGs >35 mm Hg (which may be more clinically relevant, as at that magnitude of gradient there may be more confusion with LVOT gradients associated with HOCM) as in this subgroup the mean ratio of peak/mean gradient was 3.85 (range 2.68 to 6.1). In 23/25 patients with HOCM, the ratio was between 2 and <3; in 23/25 patients with LVCO and ICGs >35 mm Hg, the ratio was 3 or higher.

**FIGURE 7:**
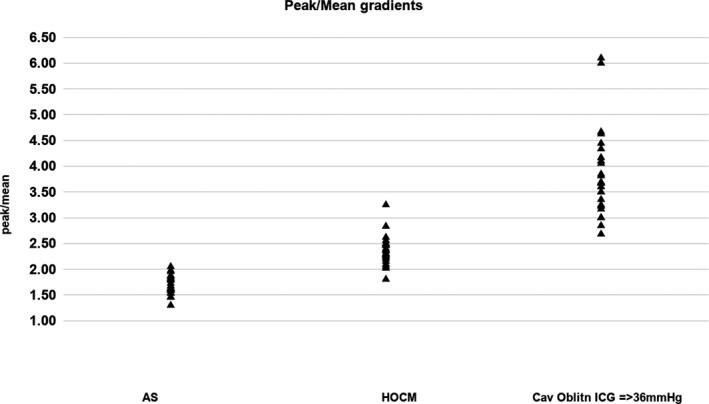
Comparison of peak/mean gradients between patients with AS, HOCM, and LVCO with ICG ≥36 mm Hg

For comparison, we assessed this ratio in patients with severe AS, and the ratio was significantly lower in this group at 1.68 (range 1.46–2.05) reflecting that the AS spectral profile is the most symmetrically parabolic contour between LVCO, HOCM, and AS. In 24/25 patients with AS, the peak/mean gradient ratio was <2 (Figure 7).

## 4 DISCUSSION

This is the first study, to our knowledge, to assess the quantitative pathophysiologic mechanism of the ICG. This is also the first study to provide a quantitative method to distinguish the LVOT spectral Doppler profile associated with HOCM from the intracavitary gradient spectral profile associated with cavity obliteration. We are not aware of any major current echocardiography textbook that details the specific nature of the intracavitary gradient associated with LVCO.

In our laboratory, approximately 1% of patients had the term “cavity obliteration” directly entered on the report (it is not currently a “check‐off” option). Most patients with LVCO, in the absence of other significant cardiac conditions, have intracavity gradients <36 mm Hg. The spectral profile associated with lower gradients differs from the patients with higher gradients. The spectral profile associated with the lower gradients is more triangular with a slow acceleration and a relatively fast deceleration. The profile of the higher gradients has an elongated fast acceleration tacked onto the initial slower acceleration, followed by a similar fast deceleration to the baseline (Figure 2).

Higher ICGs are associated with a greater *extent* of cavity obliteration, as defined by the percentage of the end‐diastolic LV cavity length obliterated in systole and more prolonged LVCO, defined as the *duration* that the LV walls are apposed. It seems logical that other factors such as the longitudinal, radial, and circumferential strain rate velocity and degree of apical twist contribute, in addition to the extent and duration of apposition, toward determining the magnitude of the gradient. As an analogy, consider the noise made when you clap your hands; it is the speed of apposing the hands as well as the surface area of hands that are apposed that correlate with the noise produced. Thirdly, higher gradients are seen in those with smaller end‐diastolic and end‐systolic cavity dimensions.

These correlations of *extent* and *duration* of LVCO with the ICG are analogous to the previously demonstrated *quantitative* and *temporal* relationships between SAM and the LVOT gradient that showed a significant correlation between the duration and timing of SAM septal contact and the LVOT gradient.^4^, ^5^ Unlike SAM‐associated gradients, which represent LVOT obstruction between the body of the LV cavity and the LVOT, the ICG gradients are presumed to arise from the gradient between the LV apex and the body of the LV beyond the virtually closed‐off apical portion of the LV. That the ICG occurs when the LV is virtually closed suggests that in these patients, the overall hemodynamic significance to the LV cavity is minimal, especially compared to LVOT gradients that occur in HOCM, while the LV is still emptying.^4^, ^5^ They do, however, imply high pressures at the LV apex.

In this regard, and to show the difference between LVOT gradients associated with HOCM and ICG gradients associated with LVCO, we studied another 25 patients with HOCM and severe SAM. We chose to compare the LVCO patients with gradients of 36 mm Hg or greater, as that level the size of the gradient lies within the realm of the gradients seen with severe SAM where the confusion may arise.

**FIGURE 8:**
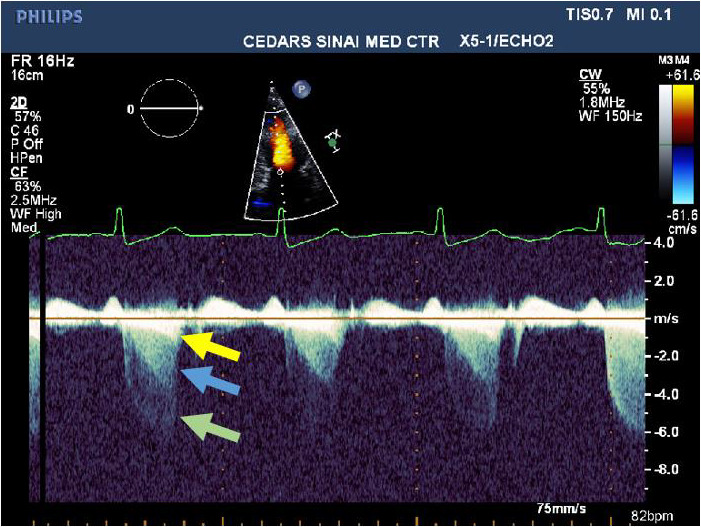
Spectral continuous‐wave Doppler profile in a patient (not in the study) with overlapping (in order of peak velocity) mitral regurgitation (green arrow), SAM‐associated LVOT obstruction (blue arrow), and LVCO (yellow arrow), demonstrating depth ambiguity

**FIGURE 9:**
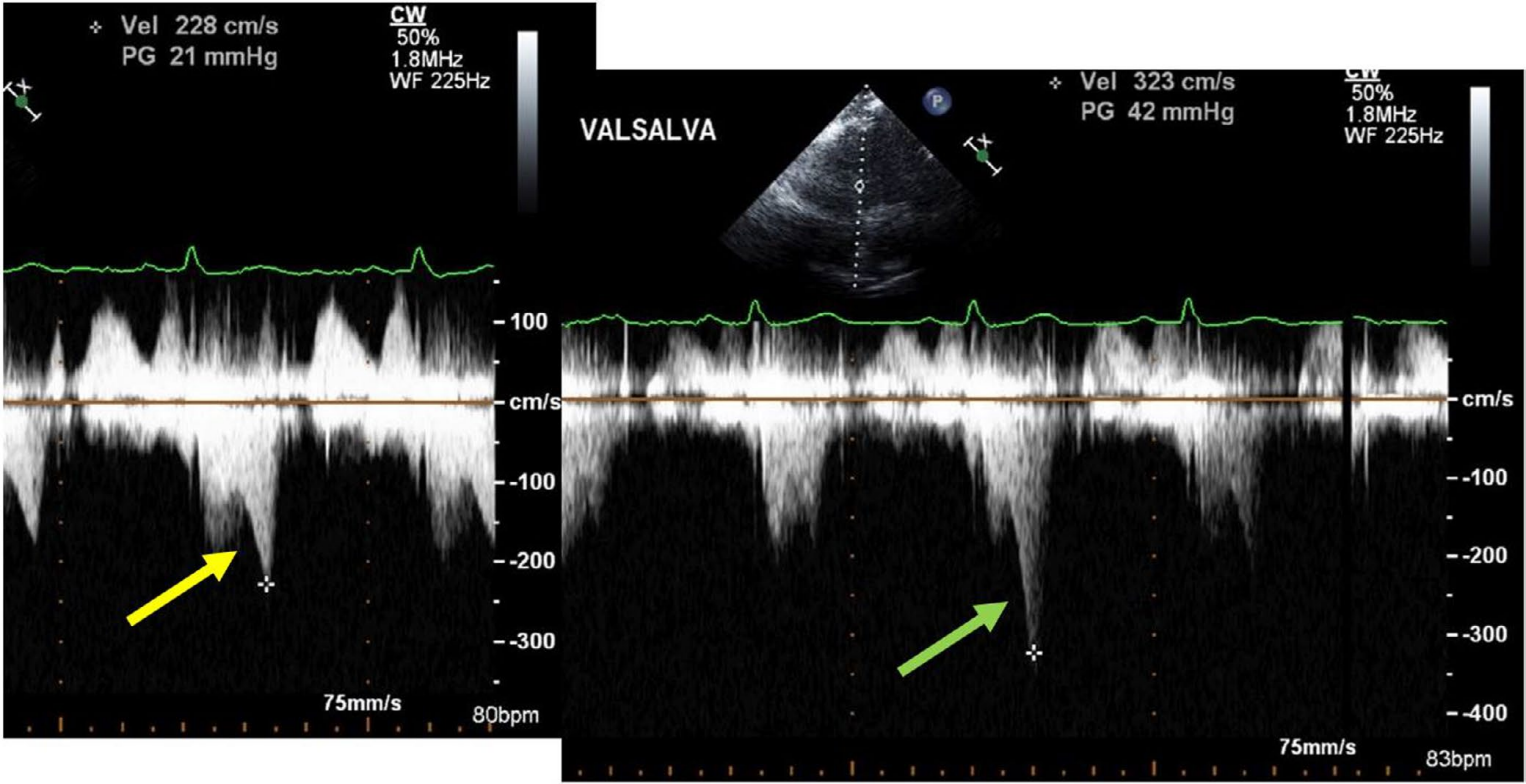
Patient with LVCO and ICG at rest (yellow arrow—left panel) that doubles with Valsalva maneuver (green arrow—right panel)

The distinction in peak/mean gradient ratios between LVCO and HOCM may be helpful to determine quantitatively the origin of a high systolic velocity obtained from the apex when the origin is uncertain or the shape of the spectral profile is ambiguous or unclear qualitatively as the ICG and LVOT spectral profiles are somewhat similar with an initial slow acceleration followed by a second faster rate of acceleration. Meticulous placement of the continuous‐wave Doppler cursor through the body of the left ventricle to separate the LVOT profile from the LVCO profile is not always possible, especially when LV cavity size is small or the cavity is not perfectly vertically aligned to the apical acoustic window. These different origin gradients may also be confused because of depth ambiguity, a known phenomenon of continuous‐wave Doppler, where spectral profiles from MR, LVOT, and ICG may overlap (Figure 8). Confusion may also arise as Valsalva maneuver increases both the ICG and the LVOT gradient (Figure 9). We have noticed that some of our less experienced sonographers may confuse LVOT spectral profiles with LVCO ICG profiles, perhaps because, in addition to the foregoing, patients with HCM and LVCO have similar hyperdynamic left ventricular contraction. The obstructed LVOT gradient spectral profile in HOCM has been likened to a dagger, presumably due to its pointed appearance. Daggers, however, have many different shapes (Figure 6), and this is an imprecise way of identifying a profile, especially as the ICG spectral profile, also has a “point,” and resembles a dagger. The ICG gradient, particularly in those with a peak gradient of more than 35 mm Hg, however, has a distinctive appearance and resembles an inverted skateboard half‐pipe slope (Figure 6). The quantitative index of separating the contours as described by the peak/mean ratio may be especially helpful clinically when the origin of the high velocity is in doubt. The distinction has clinical relevance, because treating the ICG gradient as if it were an LVOT gradient associated with HOCM,^11^ with measures such as disopyramide, septal ablation, or surgical myectomy, would be inappropriate, and potentially harmful although there is one case report^12^ of using cibenzoline to reduce the intracavitary gradient from 65 to 35 mm Hg with improvement in dyspnea.

The lower peak/mean gradient ratio for HOCM patients (2.4) than for the LVCO patients (especially those with peak gradients equal to or more than 36 mm Hg) (3.8) lends weight to the known hemodynamic and clinical significance of LVOT gradients. For example, a peak HOCM LVOT gradient of 64 mm Hg translates to a mean gradient of approximately 27 mm Hg, whereas a peak ICG gradient of 64 mm Hg is equivalent to a mean gradient of 17 mm Hg.

As a further comparison, as a contrast, and because depth ambiguity may overlay systolic velocity profiles obtained from the apex simultaneously, we also looked at 25 patients with severe AS. The peak/mean gradient ratio was lowest in this group, at 1.7, consistent with the more symmetrically shaped parabolic contour (Figure 6) associated with the AS spectral profile. For an equivalent peak gradient of 64 mm Hg, there would be a mean gradient of 38 mm Hg.

The clinical relevance of these intracavitary gradients is uncertain. It seems plausible that the higher intracavitary gradients may have significance, as the resulting high apical pressures, and the potential accompanying apical ischemia,^9^ provide a possible reason for the association between LVCO and adverse outcomes of the combined endpoint of sudden death and potentially lethal arrhythmic events, in patients who also have HOCM.^13^ These high apical pressures are also the presumed etiology for the development of apical aneurysms in patients with HOCM and coexisting LVCO.^14^ Furthermore, intense catecholamine excess, which may produce severely high apical pressures secondary to LVCO, may be the cause of the apical wall‐motion abnormality seen in Takotsubo cardiomyopathy^15^ and the LV wall‐motion abnormalities associated with subarachnoid hemorrhage.^16^

## 5 CONCLUSION

Our study provides insight into the mechanism of the ICG in patients with LVCO. Just as SAM is not an all‐or‐none phenomenon, ranging from late and minimal septal contact, which produces a small LVOT gradient, to early and prolonged septal contact that produces a large LVOT gradient,^4^, ^5^ so too LVCO is not an all‐or‐none phenomenon. Greater extent and longer duration of obliteration are associated with higher intracavitary gradients.

Our study also highlights the different qualitative differences between the Doppler spectral profiles of HOCM and LVCO. For the first time, this study reports a quantitative method of differentiation by using the ratio of peak/mean gradient. The difference between the profiles has clinical implications as the ICG associated with LVCO may be confused with the LVOT gradient of HOCM, and treatment for the former as if it were the latter would be inappropriate and potentially harmful.

### 5.1 Limitations of the study

This is a retrospective study. A prospective study performing simultaneous echocardiographic and Doppler studies would provide more insight into the pathophysiology between LVCO and the resulting ICG. Three‐dimensional echocardiographic assessment of apical obliteration might provide more accurate spatial quantification of the degree of obliteration. In addition, strain measurements of LV shortening would likely be illuminating.
